# Supplementary material for: APOBEC mutagenesis is a common process in normal human small intestine
Source: Nat Genet. 2023 Jan 26;55(2):246–54. doi: 10.1038/s41588-022-01296-5 (PMC9925384; doi:10.1038/s41588-022-01296-5)

nature genetics

Article https://doi.org/10.1038/s41588-022-01296-5

# APOBEC mutagenesis is a common process in normal human small intestine

In the format provided by the authors and unedited

**SUPPLEMENTARY INFORMATION CONTENTS**

**Supplementary Information Fig. 1 | *APOBEC1/APOBEC3A/APOBEC3B* mRNA expression across normal tissues.**

**Supplementary Information Fig. 2 | Extended contexts of APOBEC signatures.**

**Supplementary Information Fig. 3 | HDP signatures from *de novo* extraction.**

**Supplementary Information Fig. 4 | Cosine similarity plot for HDP components and shortlisted PCAWG reference signatures.**

**Supplementary Information Figure 1 | APOBEC1/APOBEC3A/APOBEC3B mRNA expression across normal tissues.**

a-b. APOBEC1 bulk tissue gene expression data. Image credit: Human Protein Atlas (v21.proteinatlas.org. a-b: https://v21.proteinatlas.org/ENSG00000111701-APOBEC1/tissue, c-d: https://v21.proteinatlas.org/ENSG00000128383-APOBEC3A/tissue, e-f: https://v21.proteinatlas.org/ENSG00000179750-APOBEC3B/tissue). (a) APOBEC1 expression levels from the Human Protein Atlas project. Duodenum nTPM = 29, small intestine nTPM = 26, colon nTPM = 0.6. (b) APOBEC1 expression levels from GTEx. Small intestine nTPM = 11.9, colon nTPM = 1.1. c-d. APOBEC3A bulk tissue gene expression data. (c) APOBEC3A expression levels from The Human Protein Atlas project. Duodenum nTPM = 1.4, small intestine nTPM = 0.8, colon nTPM = 1.5. (d) APOBEC3A expression levels from GTEx. Small intestine nTPM = 0.8, colon nTPM = 1.4. e-f. APOBEC3B bulk tissue gene expression data. (e) APOBEC3B expression levels from The Human Protein Atlas project. Duodenum nTPM = 7.3, small intestine nTPM = 4.3, colon nTPM = 12.1. (f) APOBEC3B expression levels from GTEx. Small intestine nTPM = 2.1, colon nTPM = 6.0.

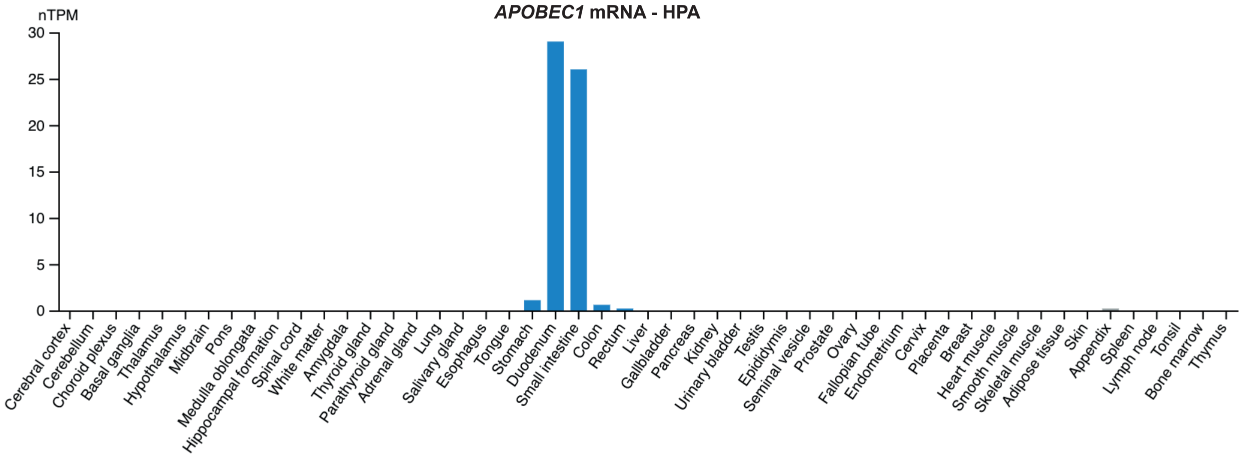


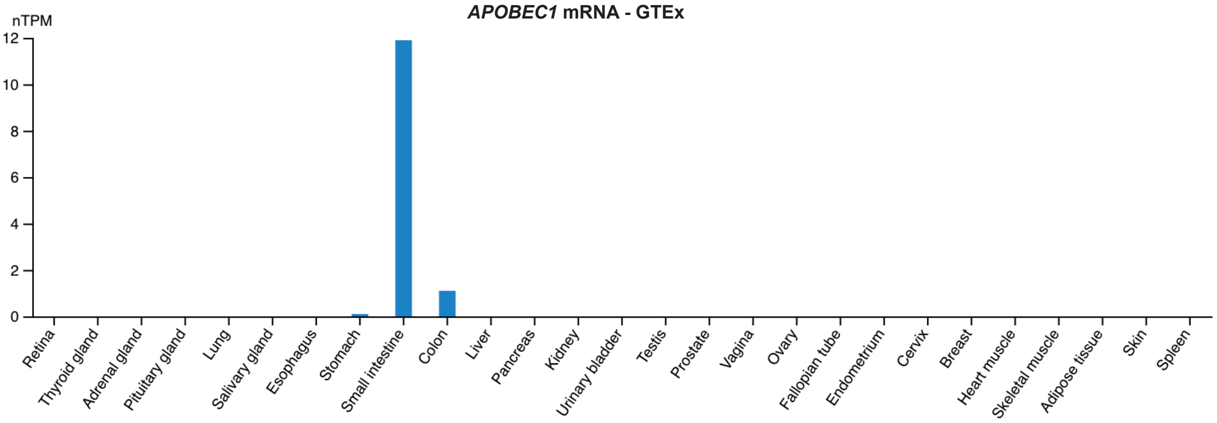


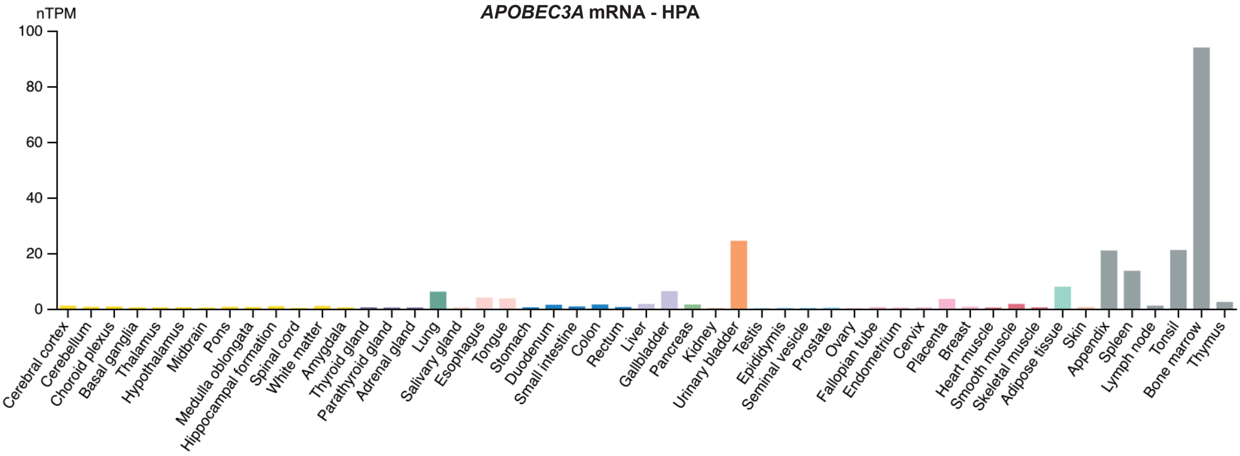

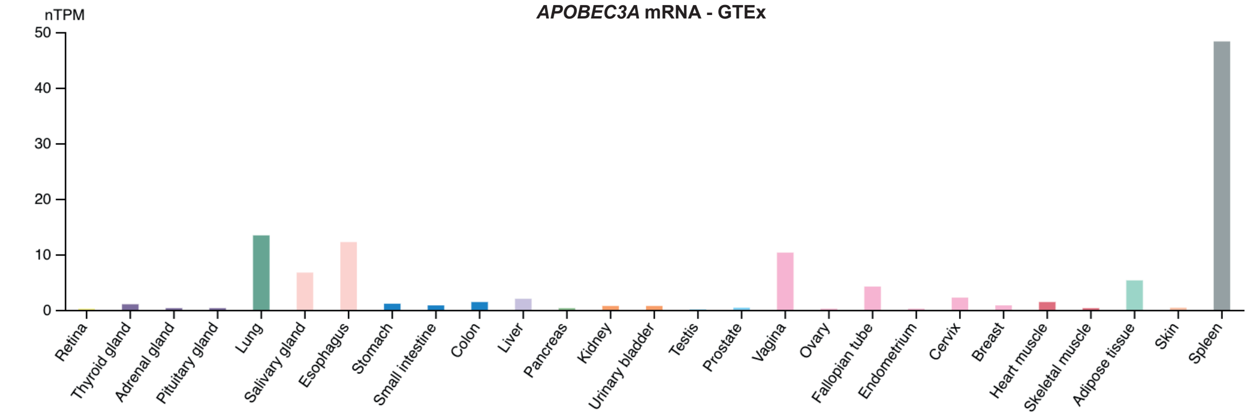
APOBEC3A mRNA - GTEx
nTPM
50
40
30
20
10
0
Retina
Thyroid gland
Adrenal gland
Pituitary gland
Lung
Salivary gland
Esophagus
Stomach
Small intestine
Colon
Liver
Pancreas
Kidney
Urinary bladder
Testis
Prostate
Vagina
Ovary
Fallopian tube
Endometrium
Cervix
Breast
Heart muscle
Skeletal muscle
Adipose tissue
Skin
Spleen

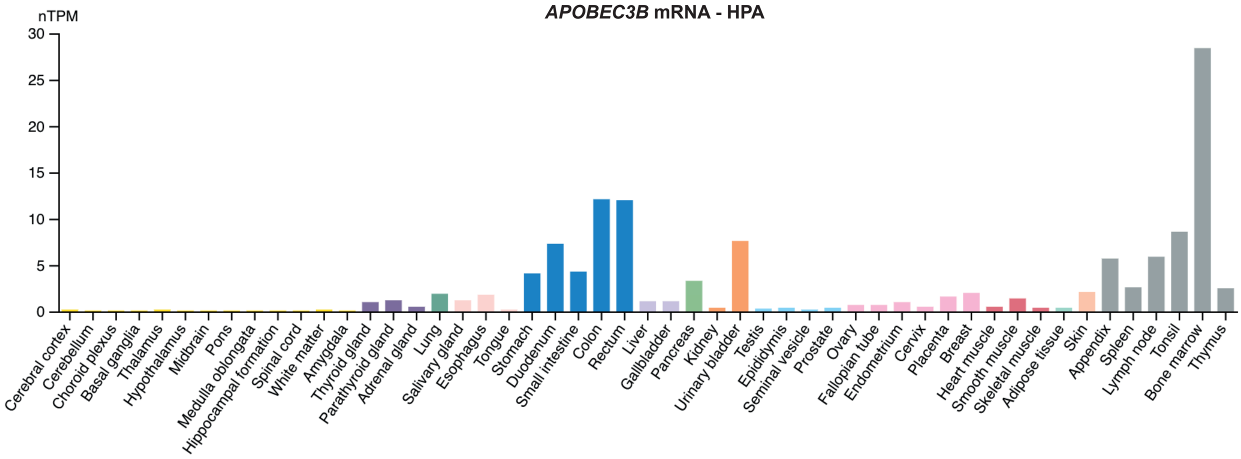
APOBEC3B mRNA - HPA
nTPM
30
25
20
15
10
5
0
Cerebral cortex
Cerebellum
Choroid plexus
Basal ganglia
Thalamus
Hypothalamus
Midbrain
Pons
Medulla oblongata
Hippocampal formation
Spinal cord
White matter
Amygdala
Thyroid gland
Parathyroid gland
Adrenal gland
Lung
Salivary gland
Esophagus
Tongue
Stomach
Duodenum
Small intestine
Colon
Rectum
Liver
Gallbladder
Pancreas
Kidney
Urinary bladder
Testis
Epididymis
Seminal vesicle
Prostate
Ovary
Fallopian tube
Endometrium
Cervix
Placenta
Breast
Heart muscle
Smooth muscle
Skeletal muscle
Adipose tissue
Skin
Appendix
Spleen
Lymph node
Tonsil
Bone marrow
Thymus

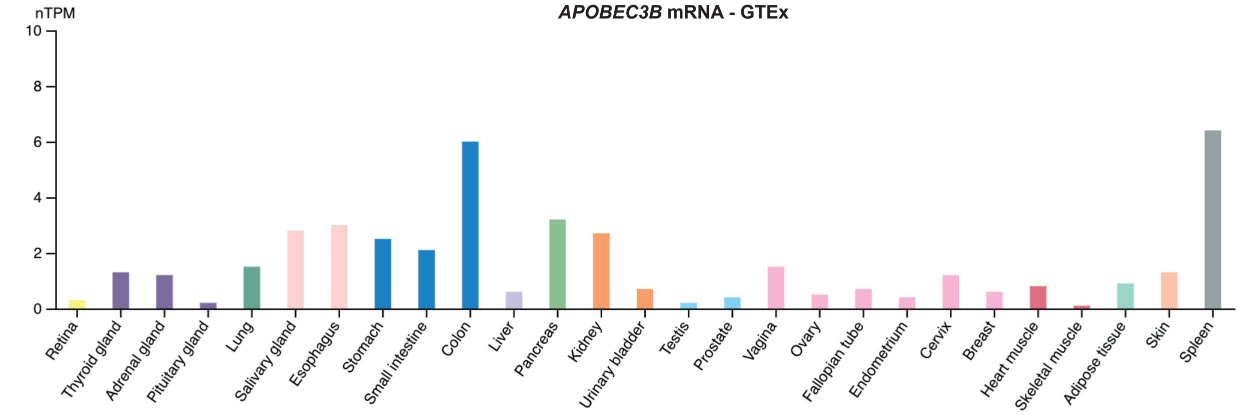
APOBEC3B mRNA - GTEx
nTPM
10
8
6
4
2
0
Retina
Thyroid gland
Adrenal gland
Pituitary gland
Lung
Salivary gland
Esophagus
Stomach
Small intestine
Colon
Liver
Pancreas
Kidney
Urinary bladder
Testis
Prostate
Vagina
Ovary
Fallopian tube
Endometrium
Cervix
Breast
Heart muscle
Skeletal muscle
Adipose tissue
Skin
Spleen

**Supplementary Information Figure 2 | Extended contexts of APOBEC signatures.**
5bp context of the C>G/T part of the APOBEC signature from HDP *de novo* extraction. X axis represents 96 categories of trinucleotide context and y axis represents the proportion. The black lines indicate 95% confidence intervals. Trinucleotide contexts that are not statistically significant are shown in light grey. The peaks show C/T at the -2 position are favoured compared to A/G. Enrichment of pyrimidines instead of purines at -2 position indicate APOBEC3B is unlikely to be the major contributing enzyme, while APOBEC1/3A could not be excluded.

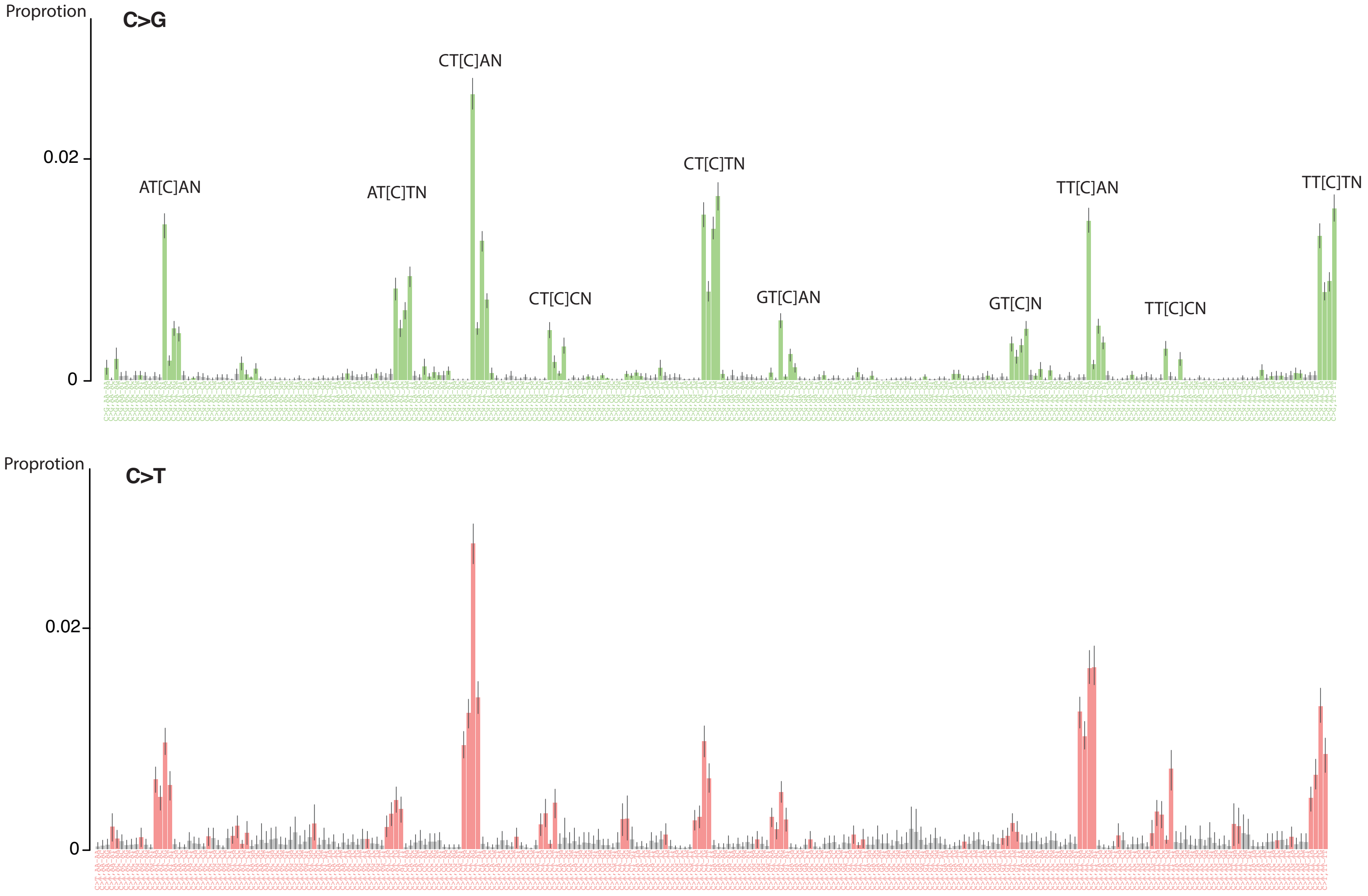

**Extended Data Fig. 9 | HDP signatures from *de novo* extraction.**
Mutational spectrum of nine HDP components from *de novo* extraction. X axis represents 96 categories of trinucleotide context and y axis represents the proportion. The black lines indicate 95% confidence intervals. Trinucleotide contexts that are not statistically significant are shown in light grey.

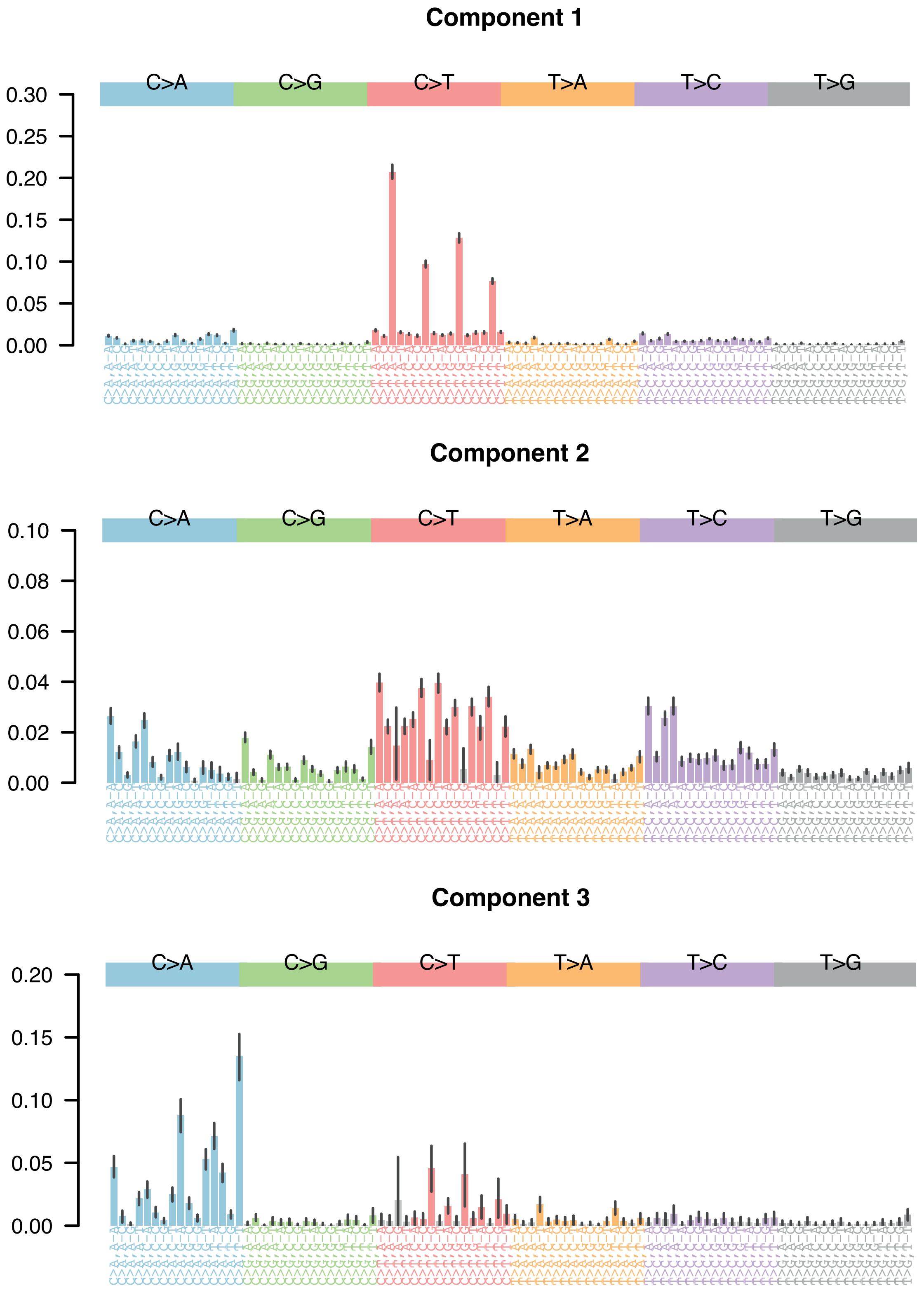

**Component 4**

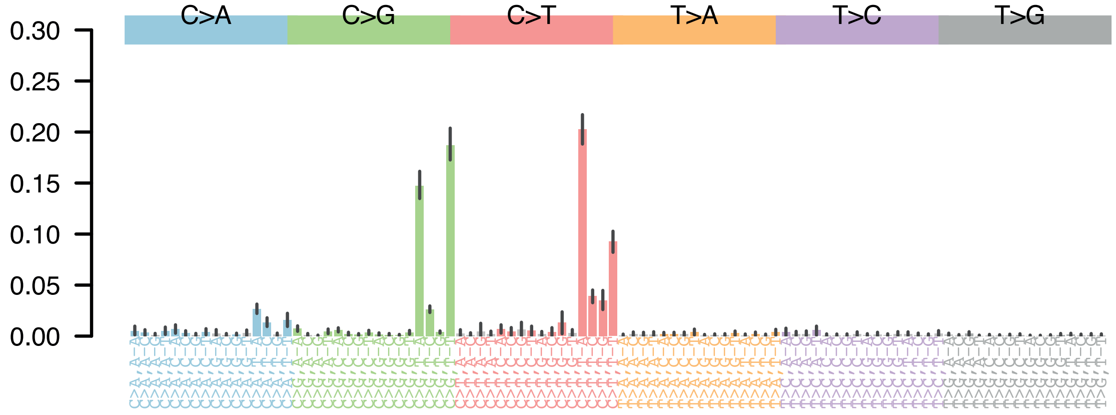


**Component 5**

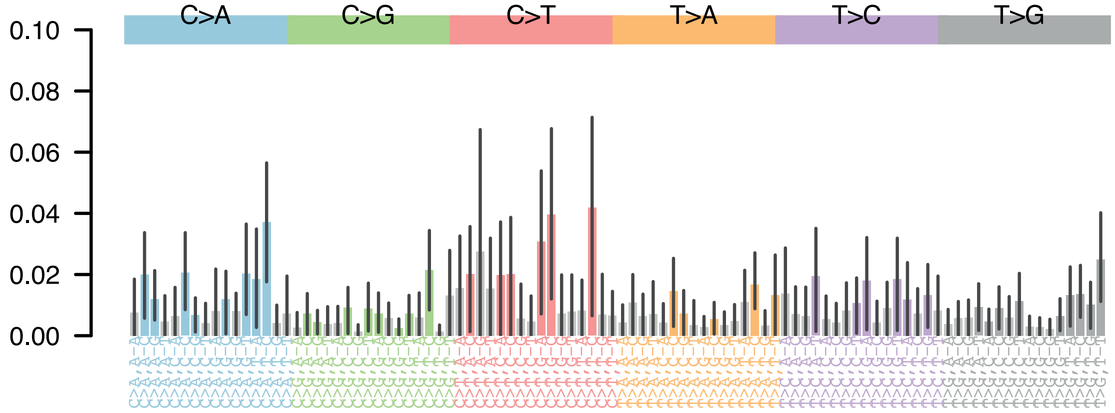


**Component 6**

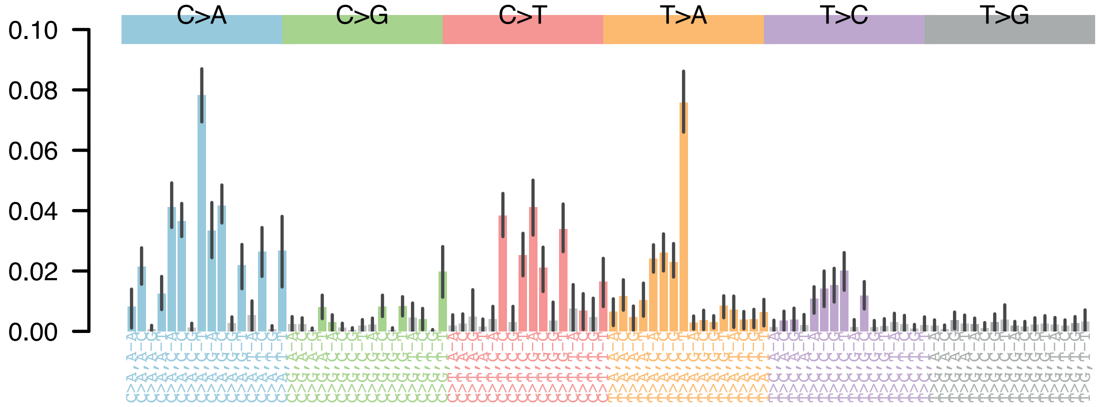

## Component 7

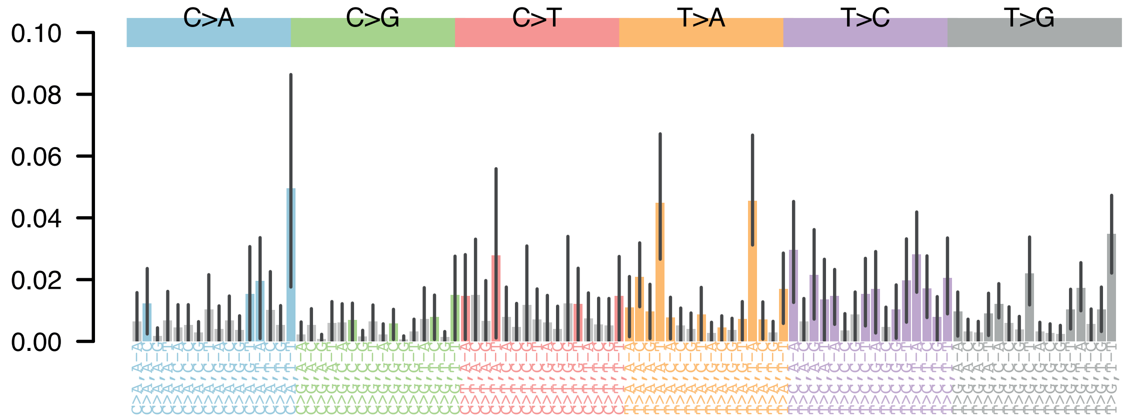


## Component 8

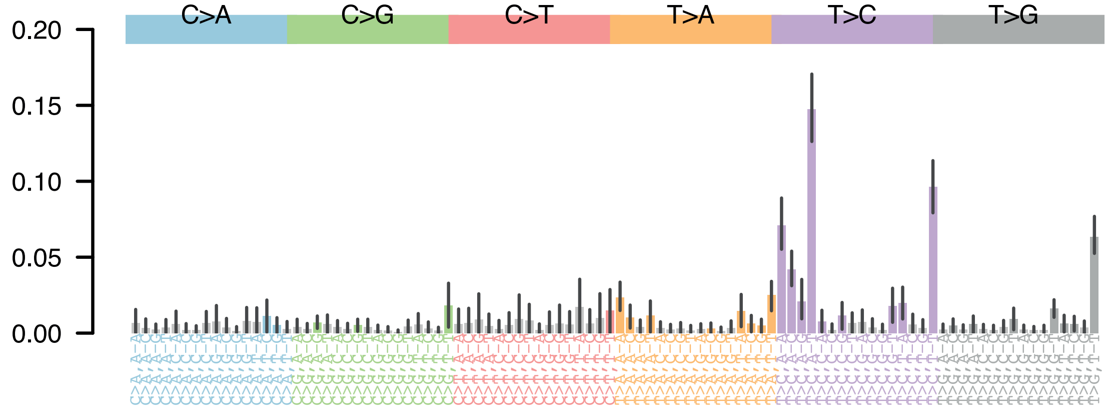


## Component 9

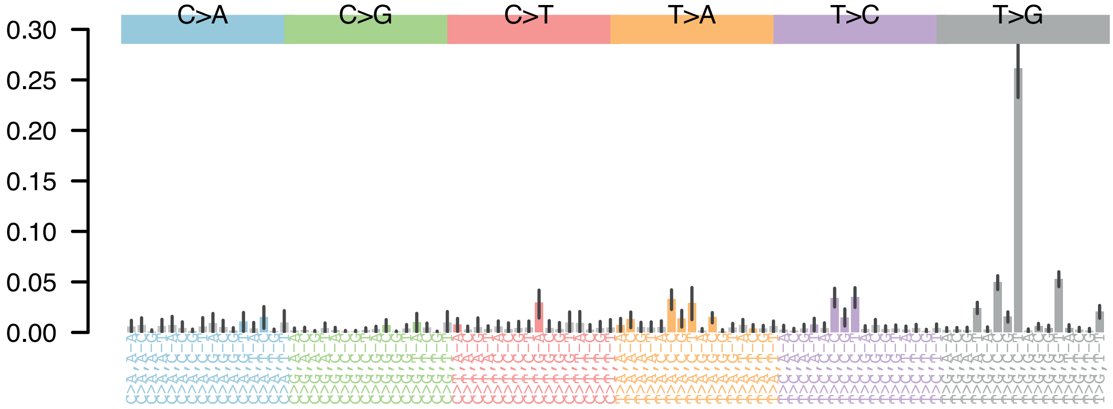

**Supplementary Information Figure 4 | Cosine similarity plot for HDP components and shortlisted PCAWG reference signatures.**
The APOBEC reference was made from the average spectrum of SBS2 and SBS13.

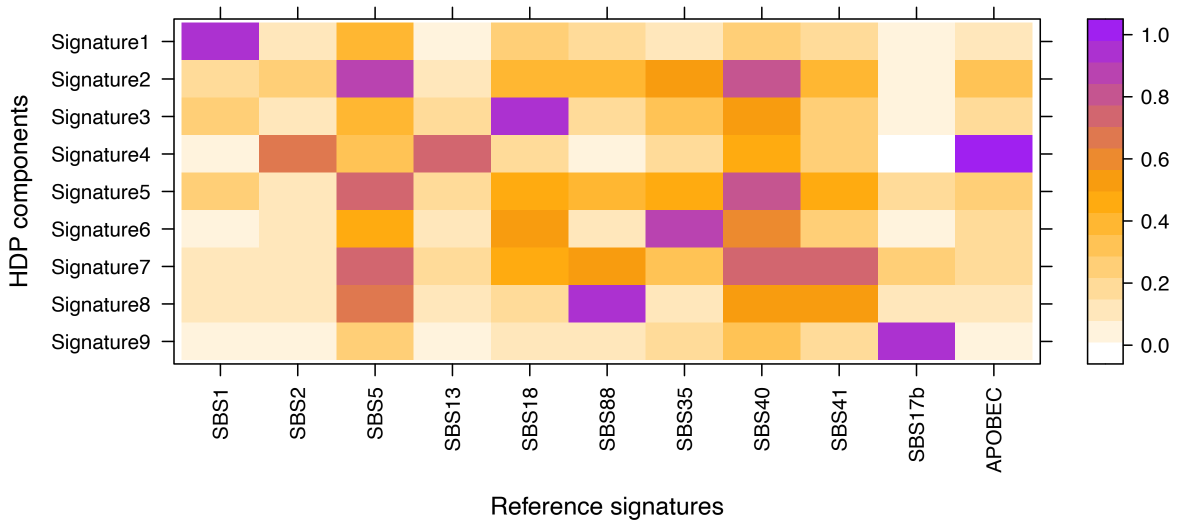

Supplement: Supplementary file 1 — Supplementary Figs. 1–4. [file 41588_2022_1296_MOESM1_ESM.pdf]
