# Supplementary material for: APOBEC mutagenesis is a common process in normal human small intestine
Source: Nat Genet. 2023 Jan 26;55(2):246–54. doi: 10.1038/s41588-022-01296-5 (PMC9925384; doi:10.1038/s41588-022-01296-5)
Supplement: Supplementary file 2 — Reporting Summary [file 41588_2022_1296_MOESM2_ESM.pdf]

nature portfolio

Corresponding author(s): Michael R. Stratton

Last updated by author(s): Nov 16, 2022

Reporting Summary

Nature Portfolio wishes to improve the reproducibility of the work that we publish. This form provides structure for consistency and transparency in reporting. For further information on Nature Portfolio policies, see our Editorial Policies and the Editorial Policy Checklist.

Please do not complete any field with "not applicable" or n/a. Refer to the help text for what text to use if an item is not relevant to your study.
For final submission: please carefully check your responses for accuracy; you will not be able to make changes later.

Statistics

For all statistical analyses, confirm that the following items are present in the figure legend, table legend, main text, or Methods section.

n/a	Confirmed	
☐	☒	The exact sample size (n) for each experimental group/condition, given as a discrete number and unit of measurement
☐	☒	A statement on whether measurements were taken from distinct samples or whether the same sample was measured repeatedly
☐	☒	The statistical test(s) used AND whether they are one- or two-sided<br>*Only common tests should be described solely by name; describe more complex techniques in the Methods section.*
☐	☒	A description of all covariates tested
☐	☒	A description of any assumptions or corrections, such as tests of normality and adjustment for multiple comparisons
☐	☒	A full description of the statistical parameters including central tendency (e.g. means) or other basic estimates (e.g. regression coefficient) AND variation (e.g. standard deviation) or associated estimates of uncertainty (e.g. confidence intervals)
☐	☒	For null hypothesis testing, the test statistic (e.g. F, t, r) with confidence intervals, effect sizes, degrees of freedom and P value noted<br>*Give P values as exact values whenever suitable.*
☐	☒	For Bayesian analysis, information on the choice of priors and Markov chain Monte Carlo settings
☐	☒	For hierarchical and complex designs, identification of the appropriate level for tests and full reporting of outcomes
☒	☐	Estimates of effect sizes (e.g. Cohen's d, Pearson's r), indicating how they were calculated

Our web collection on statistics for biologists contains articles on many of the points above.

Software and code

Policy information about availability of computer code

Data collection	No code and software was required to collect data.
Data analysis	Mutation-calling algorithms are available through GitHub (https://github.com/cancerit). Variant calling filters can be found at https://github.com/MathijsSanders/SangerLCMFiltering and https://github.com/TimCoorens/Unmatched_NormSeq .<br><br>Statistical analysis was performed in R (3.0.4 and 4.0.2). All custom code for this study is available on GitHub at https://github.com/YichenWang1/small_bowel. Any additional code will be provided by the authors on request.<br><br>Open source R packages:<br>HDP - version 0.1.5 (https://github.com/nicolaroberts/hdp)<br>sigFit - version 2.0 (https://github.com/kgori/sigfit)<br>nlme - version 3.1-148 CRAN<br>ggtree - version 3.3.1 Bioconductor<br>Seurat - version 4.1.1 CRAN<br>ape - version 5.6.1 CRAN<br>GenomicRanges - version 1.42.0 Bioconductor<br>BSgenome - version 1.58.0 Bioconductor<br>BSgenome.Hsapiens.UCSC.hg19 - version 1.4.3 Bioconductor<br>dplyr - version 1.0.7 CRAN<br>tidyr - version 1.2.0 CRAN<br>tidyverse - version 1.3.1 CRAN<br>RColorBrewer - version 1.1.2 CRAN<br>ggplot2 - version 3.3.5 CRAN

knitr - version 1.37 CRAN

Additional publicly available code and software used:
BWA-Mem - version 0.7.12-r1039 and 0.7.17 (https://github.com/lh3/bwa)
CaVEMan - versions 1.11.2, 1.14.1, 1.15.1(https://github.com/cancerit)
cgpPindel - versions 2.2.2, 3.3.0 (https://github.com/cancerit)
ASCAT - versions 4.0.1, 4.1.2, 4.5.0 (https://github.com/cancerit)
Battenberg - versions 3.5.3 (https://github.com/cancerit) BRASS
- version 6.1.2, 6.3.4 (https://github.com/cancerit)
GRIDSS - version 2.13.1 (https://github.com/PapenfussLab/gridss)
JBrowse - version 1.15.2 (https://jbrowse.org/blog/2018/08/16/jbrowse-1.15.2-maintenance-release-index.html)
MPBoot - version 1.1.0 (https://github.com/diepthihoang/mpboot)

For manuscripts utilizing custom algorithms or software that are central to the research but not yet described in published literature, software must be made available to editors and reviewers. We strongly encourage code deposition in a community repository (e.g. GitHub). See the Nature Portfolio guidelines for submitting code & software for further information.

Data

Policy information about availability of data

All manuscripts must include a data availability statement. This statement should provide the following information, where applicable:
- Accession codes, unique identifiers, or web links for publicly available datasets
- A description of any restrictions on data availability
- For clinical datasets or third party data, please ensure that the statement adheres to our policy

DNA sequencing data generated for this study are deposited in the European Genome-Phenome Archive (EGA) with accession code EGAD00001008764. There is no restriction on data availability. Existing DNA sequencing data used in study are deposited in EGA with accession code EGAD00001004192 (PD37449, PD34200, PD37266) and EGAD00001006641 (PD28690, PD43850, PD43851). Existing RNA sequencing data were downloaded from Gut Cell Survey (https://www.gutcellatlas.org/), Tabula Sapiens (https://tabula-sapiens-portal.ds.czbiohub.org/), and Gene Expression Omnibus (GSE125970 and GSE116222, read counts and cluster results downloaded from the Human Protein Atlas: https://www.proteinatlas.org/about/download).
The cBioPortal MutationMapper database used to annotate cancer hotspot mutations was accessed at:
https://www.cbioportal.org/mutation_mapper?standaloneMutationMapperGeneTab=ATM.

Field-specific reporting

Please select the one below that is the best fit for your research. If you are not sure, read the appropriate sections before making your selection.

☒ Life sciences ☐ Behavioural & social sciences ☐ Ecological, evolutionary & environmental sciences

For a reference copy of the document with all sections, see nature.com/documents/nr-reporting-summary-flat.pdf

Life sciences study design

All studies must disclose on these points even when the disclosure is negative.

Sample size	No sample-size calculation was performed. Sample size was determined by the availability of tissue and cost of the experiment.
Data exclusions	One crypt (PD42835_lo0032) was excluded from the start due to clear evidence of contamination. All other samples were included in downstream analysis and had their mutational landscape reported. When modelling mutation rates, only samples with >15-fold coverage were included, and one patient (PD43853) with a substantial number of mutations due to chemotherapy was excluded. Two biopsies (PD43851j_P52_DDM_E2, PD46565c_lo0009) have a mutational landscape dominant by SBS5/40 and with lower mutation rates, which is distinct from the mutational landscape of normal small intestinal crypts, and similar to that of Brunner's glands, despite their crypt-like appearance under microscopy inspection. These two samples was kept and reported for the comprehensiveness and transparency of this dataset, but were not included in the statistical modeling, and we ran a separate signature extraction on the remaining crypts, to avoid interference from the two unusual cases.<br>When modelling clonal dynamics, crypts from coeliac patients, children and the patient with excessive amount of mutations from chemotherapy (PD43853) were excluded, because these crypts might have different number of cells and non-constant mutation rate.
Replication	Our manuscript describes the mutational landscape of the normal tissues, it does not test specific hypotheses, and so replication does not apply in its usual way. Sequencing replicates are not normally possible as most of these samples have been obtained from distinct crypts, though mutation rates and signatures for crypts from the same individual demonstrated good concordance. Validation of the low-input whole genome sequencing protocol is detailed in Ellis et al 2021, and the protocol was applied in previously published work. Validation of low-input whole genome sequencing after crypt isolation is available as part of the studies published in Lee-Six et al 2019 and Moore et al 2020. Ellis et al 2021.
Randomization	Not applicable - cases were not subjected to any intervention. Covariates such as age, biopsy location and disease condition were controlled in statistical modelling.
Blinding	Not applicable - cases were not subjected to any intervention and were not allocated into groups. For pre-existing conditions, it was not

Blinding | possible for the researchers to remain fully unaware of the conditions, because they were self-explanatory during the collection and analysis: for the coeliac samples, pathological morphology such as disruption of villi structures were observed during laser capture microdissection; for patients undergone chemotherapy, this was revealed by chemo-therapy related mutational signatures left on their genomes.

Reporting for specific materials, systems and methods

We require information from authors about some types of materials, experimental systems and methods used in many studies. Here, indicate whether each material, system or method listed is relevant to your study. If you are not sure if a list item applies to your research, read the appropriate section before selecting a response.

Materials & experimental systems

n/a	Involved in the study	
☒	☐	Antibodies
☒	☐	Eukaryotic cell lines
☒	☐	Palaeontology and archaeology
☒	☐	Animals and other organisms
☒	☐	Human research participants
☐	☒	Clinical data
☒	☐	Dual use research of concern
☒	☐	

Methods

n/a	Involved in the study	
☒	☐	ChIP-seq
☒	☐	Flow cytometry
☒	☐	MRI-based neuroimaging

Human research participants

Policy information about studies involving human research participants

Population characteristics

The dataset includes 342 individual small intestinal crypts together with three Brunner's glands from 39 individuals (22 females and 17 males aged between 4 - 82 years from UK). Among them six individuals (two females, four males) had a history of coeliac disease (gluten enteropathy). Information for each individual is provided in Supplementary table 6.

Recruitment

Participants were recruited from eight cohorts, including organ donors (providing duodenum, jejunum and ileum samples), patients who have undergone endoscopy (providing duodenum samples) and colorectal cancer patients who have had surgical removal for part of their colon (including part of the ileum). Samples were selected to cover a wide age range and no other selection criteria were applied. In retrospect, we have a good balance of gender (22 females, 17 males). However, all participants were based in UK, and this might need to be considered when generalise the conclusions to other regions and ethic groups. To be specific, our first cohort consists of samples from a 78-year-old man (PD28690), a 54-year-old woman (PD43850) and a 47-year-old man (PD43851) in warm autopsies within 6h of death (REC 13/EE/0043 and 17/LO/1801). Samples in the second cohort were from organ donors (two females, one male) at age 36 to 67 from whom small-intestinal biopsies were taken at the time of organ donation (REC 15/EE/0152). The third cohort represents four female and three male patients aged 38 to 80 who underwent surgical resection (REC 15/WA/0131). The fourth represents four female patients aged 53 to 77 who had endoscopy (REC 08/h0304/85+5). The fifth cohort are children between 4 to 13 (four females, two males) who had endoscopy (REC 17/EE/0265). The sixth cohort comprised six male and two female patients aged 50 to 82 with surgical resection (REC 20/NW/0001). The seventh cohort are two female and four male patients aged from 37 to 78 with coeliac conditions, and samples were collected through endoscopy (REC 18/ES/0133). The last cohort are from AMSBio (commercial supplier), samples for donors PD52486, PD52487 were obtained at autopsy from two female individuals who had died of causes not related to cancer (REC 17/LO/1801).

Ethics oversight

National Research Ethics Service Committee East of England: PD28690, PD37449, PD34200, PD37266
London-Surrey Research Ethics Committee: PD43850, PD43851, PD52486, PD52487
Wales REC 7: PD41851, PD41852, PD41853, PD42833, PD42834, PD42835, PD43853
NRES Committee East of England – Cambridge East: PD43400, PD43401, PD43402, PD43403
NRES Committee East of England – Cambridge South: PD43949, PD43950, PD43951, PD43952, PD43953, PD43954
NRES Committee North West – Haydock: PD45766, PD45767, PD45769, PD45770, PD45771, PD45773, PD45776, PD45778
East of Scotland Research Ethics Service : PD46562, PD46563, PD46565, PD46566, PD46568, PD46573

Note that full information on the approval of the study protocol must also be provided in the manuscript.

This checklist template is licensed under a Creative Commons Attribution 4.0 International License, which permits use, sharing, adaptation, distribution and reproduction in any medium or format, as long as you give appropriate credit to the original author(s) and the source, provide a link to the Creative Commons license, and indicate if changes were made. The images or other third party material in this article are included in the article's Creative Commons license, unless indicated otherwise in a credit line to the material. If material is not included in the article's Creative Commons license and your intended use is not permitted by statutory regulation or exceeds the permitted use, you will need to obtain permission directly from the copyright holder. To view a copy of this license, visit http://creativecommons.org/licenses/by/4.0/
